# Sit, stand, and swivel: Posture affects visual exploration of panoramic scenes in virtual reality

**DOI:** 10.1371/journal.pone.0334182

**Published:** 2025-10-28

**Authors:** Avi Mehrotra, Crystal Silver, Walter F. Bischof, Alan Kingstone

**Affiliations:** 1 Department of Psychology, University of British Columbia, Vancouver, Canada; 2 Department of Psychology, University of Aberdeen, Aberdeen, Scotland; Gachon University, KOREA, REPUBLIC OF

## Abstract

This 45-minute study, composed of 27 participants (20 female, 7 male) from the University of British Columbia (mean age 21.5 years), systematically examined how posture -- sitting in a stationary chair, standing, or swiveling in a chair -- affects visual exploration of immersive virtual environments. Using 360° panoramic scenes, we analysed eye, head, and torso movements to assess the spatial extent and coordination of visual behavior. Standing posture enabled the greatest movement range and scene coverage, while fixed sitting constrained exploration, resulting in compensatory eye-in-head activity. The swivel condition closely approximated standing, suggesting that rotational freedom, not upright posture alone, drives naturalistic gaze behavior. Analyses confirmed that posture significantly shapes horizontal movement distribution, especially for head and torso. Eyes led head and torso movements, revealing a dynamic, nested coordination pattern. These findings, based on the unique integration of high-precision oculomotor data with a systematic comparison of different postures, extend prior work and emphasise posture’s critical role in shaping embodied vision in virtual reality. Beyond research design implications, our results inform VR-based physical therapy and immersive skill training, highlighting the need to consider physical movement affordances in immersive contexts.

## Introduction

Early eye movement research in lab settings often used methodologies involving head immobilization and preselected stimuli consisting of static images, text, or videos presented on computer monitors [1,2]. This approach provides high measurement precision and experimental control, making it suitable for studying basic visual processes. However, the artificial constraints of head immobilization and screen-based stimuli limit the ecological validity and generalizability to natural gaze behaviours. In everyday settings, people typically move their heads to explore their environment, and these movements alter both what information is accessed [3] and how it is encoded and remembered [4].

Mobile eye trackers ‘in the wild’ partially address limitations by allowing free movement of the head and body; though they can fall short in other respects. These systems track the eyes relative to the head (i.e., head-centred) and head position is estimated through scene-camera alignment or external sensors [5]. Accurately determining head position in world coordinates is challenging due to the visual dynamic nature of the observer and the environment. This can compromise measurement accuracy, making it difficult to assess the interaction between eye and head movements.

Eye tracking in virtual reality (VR) can address these shortcomings while maintaining ecological validity. VR allows precise control and manipulation of fully immersive 360° visual environments, along with accurate recording of eye and head position in world-based coordinates, enabling detailed analysis of their interaction. Furthermore, VR systems can integrate external motion trackers or inertial measurement units (IMUs) to track body position relative to the head and eyes. As such, VR-based eye tracking offers an effective compromise between ecological validity and experimental control.

### Posture and VR

One of the fundamental decisions that one must make when conducting VR research is how to position a user (see [6] for an excellent comprehensive consideration of VR protocols, and for a historical perspective see [7]). Specifically, should they be standing or seated, and if they are seated should the chair swivel or not? While standing, participants are free to move their body and head naturally. Seated in a swivel chair, individuals can rotate along the horizontal axis by swiveling, without recruiting much head or torso movement. And while sitting in a fixed chair, they are restricted from moving their feet to reorient their bodies. In sum, different postures change the biomechanics of eye-head-torso coordination, and with it, the nature of visual exploration itself.

To our knowledge, there is no consensus among researchers that have studied visual exploration in VR regarding how participants should be physically positioned, nor the impact of different postures on performance within VR. A review of the methodology of the existing studies suggests that posture is often either overlooked, not a significant consideration in design, or a secondary consideration. In our past work using panoramic natural scenes, we have placed participants in a swivel chair [8] and a stationary chair [9]. Other VR studies using panoramic scenes have used swivel chairs [10], and had participants stand, or mixed sitting and standing across different conditions [11]. Indeed, several studies have not specified the posture that was used, though sometimes one can infer the posture of the participants [12,13].

Sidenmark and Gellersen’s recent study [14] is a notable exception. They examined how eyes, head, and torso coordinate in different postures -- standing and sitting in a stationary (i.e., non-swivel) chair -- to facilitate gaze shifts in VR. Participants had to visually locate a single target within a hemispherical virtual environment radiating from the participants’ central position when facing forward. Targets could be presented either within or outside the participants’ field of view (FOV); for targets outside the FOV, an arrow directed participants towards the target location. Participants showed a preference for supporting head movements with torso rotation in the standing condition, even when the head movement was well within head motion range. When this head-torso relationship was more constrained (i.e., in the fixed seated condition) the head-in-torso range increased significantly, meaning the head moved more relative to the torso to expand the FOV. Interestingly, the contribution of the eyes for gaze shifts did not differ between postures.

Despite Sidenmark and Gellersen’s excellent contribution [14], there are some outstanding questions. Because their study used a sparse environment -- where movements were directed either to a single target within a blank visual field or to a target outside the FOV indicated by an arrow -- it remains unclear how participants would explore rich visual environments without a predefined goal, as often happens in everyday life. Additionally, stimuli were displayed within a range of 5–100º from a central fixation point, equivalent to a 200º field of regard. The question of how people coordinate their movements in a fully immersive omnidirectional 360º environment remains unanswered. Finally, the question of performance in a swivel-chair was not investigated. In sum, a systematic investigation of the effects of posture (standing, swiveling, sitting in a stationary chair) on the mechanics of eye, head, torso movements and their coordination during visual exploration of a rich visual environment has yet to be conducted.

### Present study

In the present study, we asked participants to visually explore 360° panoramic scenes in VR in three different postural conditions: standing, seated in a swivel chair, and seated in a stationary chair. In all conditions, the participants’ eye movements, head movements, and torso movements were tracked and recorded. The swivel chair’s rotation was also tracked. Our focus was on understanding how the biomechanical alterations (restricting or facilitating movement) induced by each postural condition influence visual exploratory behaviour. Specifically, we investigated the relationship between eye, head, torso and body movements and the differential recruitment of each effector in enabling visual orientation as affected by posture in VR. Our primary research questions were:

1. How does posture influence gaze behaviour during visual exploration of static panoramic scenes in VR?
2. How does each effector -- defined in the present study as eye, head, and torso -- move to facilitate gaze behaviour observed in each postural condition (i.e., Sit, Stand, or Swivel). And what are the relative contributions of each effector in facilitating the observed gaze behaviour?
3. What is the temporal order of relative effector movements? Is there an effector recruitment preference?

## Methods

### Participants

27 participants were recruited (20 female, 7 male, aged from 18–38 years, mean age = 21.5 years) from the University of British Columbia Department of Psychology SONA Human Subject Pool (09/09/2024-01/03/2025) and participated in the experiment in exchange for course credit. All participants reported normal or corrected-to-normal vision and if needed, were told to wear contact lenses instead of glasses for the experiment. Written consent was obtained from all participants, and all experimental procedures and protocols were reviewed and approved by the University of British Columbia’s Behavioural Research Ethics Board (H22-00572).

### Design

Our experiment had one within-subjects factor of ‘Posture’ with three conditions: sitting in a stationary chair with armrests (i.e., sit), sitting in a swivel chair with armrests (i.e., swivel), and standing (i.e., stand). The only difference in these conditions was the presence of a chair and the type of chair.

### Materials and apparatus

Stimuli. The stimuli used throughout were 360° panoramic scenes projected onto a sphere that surrounded the participant in the virtual space, such that the participant appeared to be immersed in the scene. The scenes consisted of 96 full 360° panoramic images with a resolution of 4096 x 2160 pixels, taken from the SUN360 Panorama Database [15]. The content of the images was equally divided to include outdoor landscapes and indoor scenes (e.g., restaurants, museums, churches).

Hardware. The stimuli were presented on an HTC Vive virtual reality headset (Vive model 2PU6100) with a built-in SMI eye tracker [16]. The headset display has a monocular resolution of 1080 x 1200 pixels (and binocular resolution of 2160 x 1200 pixels), 90 Hz refresh rate, a horizontal and vertical field of view of approximately 110° and 113° respectively, and a weight of 635 g. The VR headset was powered by a custom-built desktop PC with the following specifications of components: Intel i7-8700K CPU @3.70 GHz, 32 GB RAM @ 2333 MHz, 1TB SSD, Nvidia GeForce GTX 1080 Ti graphics card. The participants were given one of the HTC Vive controllers to interact with the experiment (i.e., to change scenes); the other was placed on the back of the participant’s swivel chair to track its rotation during each trial. We also used an HTC motion tracker and strapped it to participants’ upper chest to track torso rotations. The average RMS orientation error of the HTC Vive tracker has been estimated at 1.46 ± 0.59°, and the difference between the difference between HTC Vive and Vicon trackers has been estimated at 1.61 ± 0.62° [17].

Eye movements were recorded using the built-in SMI eye tracker at a sampling rate of 250 Hz within the full field of view of 110° at an accuracy of 0.2°. Head movements were tracked at a sampling rate of 90 Hz using two infrared sensors (base stations) affixed at opposite corners of the room at a height of 8 feet. According to [18], the orientation precision of the Vive headset is on the order of 0.01° for a non-moving observer, as was the case in the present study.

Software. The virtual space for the experiment was created in the Unity game engine [19] as a ‘scene’ object. Gaze and head tracking were added to the scene using the corresponding prefab included in the SMI plugin for Unity. Stimulus presentation, timing and recording of gaze and head tracking were controlled through a custom C# script. Data analyses were done using Matlab [20] and statistical analysis were done using R [21,22].

### Procedure

Upon arrival, participants were briefed about the study procedure, requested to give their informed consent and asked three questions (i.e., age, sex and use of optical aids). They were fitted and familiarised with the VR headset and given an HTC Vive controller to interact with the experiment and initiate trials. Next, a five-point SMI eye-tracker calibration was conducted which required participants to follow a moving dot while keeping their head still. This calibration was repeated every 30 trials.

Each trial began with a fixation cross laid on a neutral grey background. During this fixation trial, a beam of light was projected from the motion tracker strapped to the participant’s chest. Participants were asked to align the beam with the cross before advancing to the image to ensure that the starting point in space was the same for each trial. Each image was shown for 10 seconds, and participants were instructed to explore each scene and try to remember its contents for a questionnaire later. This was done to encourage thorough exploration of each scene [23]. No questionnaire was delivered, with the reason for the deception explained during debriefing, in accordance with our ethics approval. 30 images were presented for every Posture condition, totalling 90 trials. The order of the conditions was counterbalanced across participants, and each image presented in those conditions was randomized.

### Definition of terms

In the following sections, we report on the analysis of head movements and eye movements within the head coordinate system, referred to as “eyes-in-head”, as well as their integration into the scene coordinate system, termed “eyes-in-space” or “gaze” [10]. The integration process involves representing head rotation with a quaternion [24] and combining it with the eyes-in-head direction vector.

**Fig 1:**
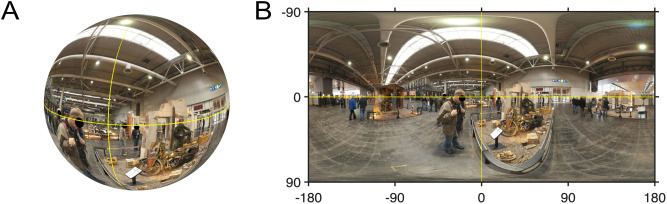
Stimulus sphere and equirectangular map. *Note*. Panoramic scene (from [15]) with equator and meridian of the initial fixation position highlighted with yellow lines. Panel A: Scene shown as the 3D projected sphere within the virtual environment; Panel B: Scene shown as an equirectangular 2D map. The map wraps around at the west meridian (−180°) and the east meridian (+180°). Note the distortions near the north and south poles, which must be taken into account when analyzing fixation patterns. In addition, while it is tempting to infer that subtle perceptual dissonances may arise for the different postures, such as a change in eye height for standing versus sitting, this in fact does not arise because the virtual sphere onto which the panoramas are projected are centred in the participants headset, independent of whether they are sitting or standing.

Participants experienced the panoramic scenes from the centre of a virtual sphere, inside which the images are projected (see Fig 1A). An “eye point” in world coordinates is defined as the location where the eye direction vector intersects with the sphere, described by longitudinal [−180°, 180°] and latitudinal [−90°, 90°] coordinates. Similarly, a “head point” in world coordinates is marked by the forward-facing vector from the face intersecting the sphere, also defined in longitude and latitude. To analyze eye points, these panoramas can be projected onto a flat equirectangular map, as depicted in Fig 1B. Distances between positions are calculated using orthodromic distances, which represent the shortest path between two points on the sphere.

In the analysis of eye movements, we focused on the detection of fixations and saccades. For the detection of fixations, we used the Dispersion-Threshold algorithm [25], which assumes that the dispersion of gaze points within a fixation is relatively small (in our study 3°) and that the duration of fixations exceeds a minimum duration (in our study 80 ms). Given a sequence of fixations, saccades were defined by the difference between successive fixations. Saccades exceeding a maximum duration (in our study 300 ms) were ignored.

## Results

### Basic eye movement metrics

Our initial analyses examined the basic eye movement metrics -- fixation number and fixation duration, as well as saccade amplitudes -- across the different postures. Although stimulus type (indoor/outdoor scene) was not of theoretical interest to us, we included it as a variable, with Posture (sit, swivel, stand) as the other variable for each eye movement metric. Data were analyzed using subject averages and repeated-measure analyses of variance (ANOVA) with factors Posture (sit, swivel, stand) and Stimulus Type (indoor, outdoor). When sphericity was violated, Greenhouse-Geisser correction was used to correct the degrees of the F-distribution [22].

The means are presented in Table 1, for each posture and each stimulus type. The mean number of fixations during the 10 seconds of viewing time varied from 45.8 to 47.7, and the fixation durations from 182.6 to 198.9 ms.

**Table 1:** Basic eye movement metrics.

Posture	Stimulus type	Number of fixations per trial	Fixation duration (ms)
Sit	Indoor	46.4	188.3
	Outdoor	46.3	198.9
Swivel	Indoor	46.6	189.1
	Outdoor	45.8	193.5
Stand	Indoor	47.7	182.6
	Outdoor	47.1	188.6

Each of these two eye movement metrics was analyzed using a subject-wise, repeated-measures ANOVA with Posture (Sit, Swivel, Stand) and Stimulus Type (Indoor, Outdoor) as factors. For the number of fixations, Posture, *F*(1.85, 48.23) = 2.93, *p* > .05, $\eta_{G}^{\text{2}}=\text{0}.\text{0}\text{1}\text{6}$, had a significant effect with the standing posture yielding the most fixations, but Stimulus Type, *F*(1, 26) = 2.86, *p* > .05, $\eta_{G}^{\text{2}}=\text{0}.\text{0}\text{0}\text{4}$ and its interaction, *F*(1.82, 47.29) = 0.29, *p* > .05, $\eta_{G}^{\text{2}}=\text{0}.\text{0}\text{0}\text{1}$ were not significant. For fixation durations, both Posture, *F*(1.93, 50.06) = 4.05, *p* < .05, $\eta_{G}^{\text{2}}=\text{0}.\text{0}\text{1}\text{8}$, and Stimulus Type, *F*(1, 26) = 13.51, *p* < .001, $\eta_{G}^{\text{2}}=\text{0}.\text{0}\text{2}\text{0}$, returned significant main effects, with standing and indoor scenes yielding the shortest durations, but again their interaction, *F*(1.71, 44.46) = 1.29, *p* > .05, $\eta_{G}^{\text{2}}=\text{0}.\text{0}\text{0}\text{3}$, was not significant.

### General visual exploration

We now turn to more detailed examinations of the way that participants explored the visual scenes. Table 2 presents the average total distance participants moved across the scenes, which is effectively a sum of the saccade amplitudes in each trial for each of the three Postures. As indicated in the table, participants covered the most distance in the stand condition (641.0°), and the least in the sit condition (569.2 to 595.0°), with the swivel condition falling in the middle (603.2 to 612.3°). A repeated-measures ANOVA returned a significant effect of Posture, *F*(1.93, 50.27) = 7.72, *p* < .001, $\eta_{G}^{\text{2}}=\text{0}.\text{0}\text{3}\text{2}$, but Stimulus type, *F*(1, 26) = 3.27, *p* > .05, $\eta_{G}^{\text{2}}=\text{0}.\text{0}\text{0}\text{2}$, and the interaction, *F*(1.99, 51.67) = 1.31, *p* > .05, $\eta_{G}^{\text{2}}=\text{0}.\text{0}\text{0}\text{2}$, were not significant.

**Table 2:** Total distance participants’ gaze moved across scenes and percentage scene exploration by fixations.

Posture	Stimulus type	Total distance (°)	Exploration (%)
Sit	Indoor	595.0	2.07
	Outdoor	569.2	2.02
Swivel	Indoor	612.3	2.12
	Outdoor	603.2	2.04
Stand	Indoor	641.0	2.18
	Outdoor	641.0	2.13

While the total distance the eyes moved was different, this does not mean that participants examined different areas of the scenes (e.g., one could reinspect an area more in one posture than another). To assess the proportion of a scene that was viewed by participants any new area within a given radius (3°) that was encompassed by a fixation on the sphere was classified as being explored, with the percentage calculated as the sum of all explored areas divided by the total area of the panoramic image, multiplied by 100 (Given a view sphere with radius r and surface area 4πr^2^, the area around each fixation within an angle θ is 2πr^2^(1-cosθ) and thus covers 50(1-cosθ) percent of the view sphere. With an angle of 3°, this amounts to 0.06852 percent of the view sphere. For multiple fixations, the overlap of adjacent fixation areas has to be taken into account). The results are also presented in Table 2. Participants explored a greater percentage of the scenes in the stand posture (2.13 to 2.18 percent) than the sit or swivel postures, with the latter two being quite similar. This was confirmed by a repeated measures ANOVA, which returned a significant effect of Posture, *F*(1.98, 51.36) = 8.13, *p* < .001, $\eta_{G}^{\text{2}}=\text{0}.\text{0}\text{2}\text{2}$, and of Stimulus type, *F*(1, 26) = 15.81, *p* < .001, $\eta_{G}^{\text{2}}=\text{0}.\text{0}\text{0}\text{8}$, with again no interaction between the two, *F*(2.00, 52.00) = 0.72, *p* > .05, $\eta_{G}^{\text{2}}<\text{0}.\text{0}\text{0}\text{1}$. Given that stimulus type is not interacting with posture, we collapse across this variable in the analyses below.

### Individual movement of the eyes, head, and torso

**Fig 2:**
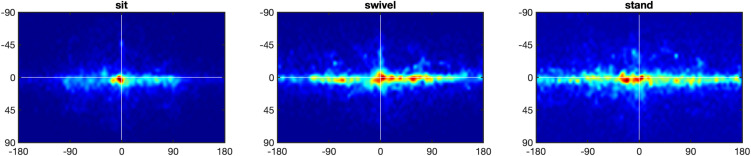
Heatmaps of eyes-in-space. *Note*. Heatmaps of eyes-in-space points in the longitude range [–180°, 180°] and the latitude range [–90°, 90°], with the white lines showing 0° longitude and latitude. Heatmaps shown for the sit condition (left), the swivel condition (middle), and the stand condition (right).

**Fig 3:**
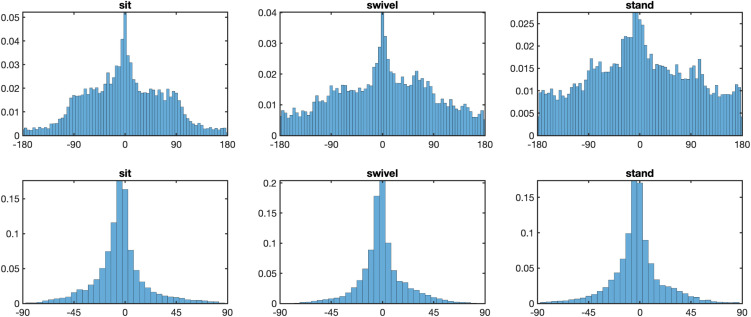
Longitude and latitude distributions of eyes-in-space. *Note.* Top panel: Longitude distributions of eyes-in-space for posture conditions sit (left), swivel (middle) and stand (right). Bottom panel: Latitude distributions of eyes-in-space for posture conditions sit (left), swivel (middle) and stand (right).

We now turn to the question of the movement of the eyes, head, and torso while participants looked at the scenes. Fig 2 presents heatmaps for eyes-in-space fixation patterns for the three postural conditions. They depict the aggregated fixation locations across all trials for all participants in our study for each of our experimental conditions with respect to where gaze lands on the sphere in world coordinates. The spread of gaze fixations in the horizontal and vertical directions are represented by longitude and latitude respectively. For all three conditions, the patterns of eyes-in-space fixations are concentrated around the equator line (i.e., latitude 0°). The top panel of Fig 3 replots the horizontal data in Fig 2 as a frequency distribution, as this form of illustration will also be used below for subsequent analyses. The spatial distribution of fixations is highly anisotropic for all three conditions. The spread is greatest in the stand condition (*SD* Longitude = 88.8°) and smallest in the sit condition (*SD* Longitude = 65.8°), with the swivel posture falling in the middle (*SD* Longitude = 83.5°). This was confirmed with a one-way ANOVA with factor Posture, *F*(1.90, 49.51) = 58.43, *p* < .001, $\eta_{G}^{\text{2}}=\text{0}.\text{2}\text{7}\text{3}$, and Bonferroni-corrected post-hoc comparisons yielding significant differences between the sit and swivel conditions (*p* < .001), the sit and stand conditions (*p* < .001), but not between the swivel and stand conditions (*p* > .05).

Though the vast amount of the movement is horizontal, the bottom panel of Fig 3 shows that the vertical spread of fixations does vary between postures, with the spread in the swivel condition (*SD* Latitude = 19.3°) being smaller than the spread in either sit (*SD* Latitude = 22.0°) or stand (*SD* Latitude = 21.8°) conditions. This was confirmed with a one-way ANOVA for posture, *F*(1.81, 47.02) = 12.19, *p* < .001, $\eta_{G}^{\text{2}}=\text{0}.\text{1}\text{2}\text{2}$, and Bonferroni-corrected post-hoc comparisons yielding significant differences between the sit and swivel conditions (*p* < .001), the swivel and stand conditions (*p* < .005), but not between the sit and stand conditions (*p* > .05).

**Fig 4:**
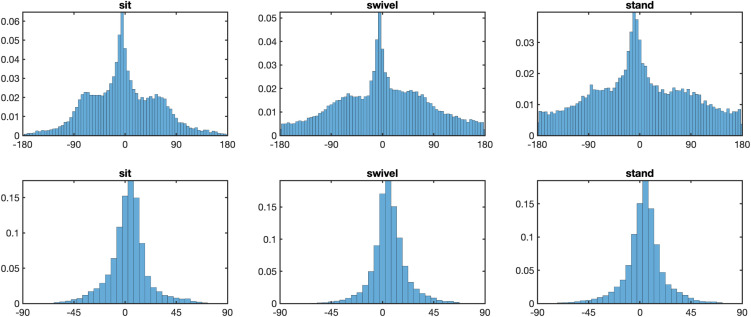
Longitude and latitude distributions of head positions. *Note.* Top panel: Longitude distributions of head positions for posture conditions sit (left), swivel (middle) and stand (right). Bottom panel: Latitude distributions of head positions for posture conditions sit (left), swivel (middle) and stand (right).

Having found that where participants look differs between the postures, the question is what role the head plays in these differences? Fig 4 presents the longitude and the latitude distributions of the head positions. As with the fixation patterns, there are significant differences in head movements across conditions, but again, primarily in the horizontal direction, with any differences in the vertical direction being relatively minor. Participants move their heads horizontally the most in the stand posture (*SD* Longitude = 82.6°), followed by the swivel posture (*SD* Longitude = 75.6°), with the least movement appearing in the sit posture (*SD* Longitude = 55.5°). The differences between the posture conditions were confirmed using a one-way ANOVA, *F*(1.91, 49.65) = 69.80, *p* < .001, $\eta_{G}^{\text{2}}=\text{0}.\text{2}\text{9}\text{7}$, and Bonferroni-corrected post-hoc comparisons yielding significant differences between the sit and swivel conditions (*p* < .001), between the sit and stand conditions (*p* < .001), and between the swivel and stand conditions (*p* < .02). With respect to the vertical distribution head distributions, a one-way ANOVA returned a significant result, *F*(1.62, 42.1) = 8.62, p < .001, $\eta_{G}^{\text{2}}=\text{0}.\text{0}\text{7}\text{6}$, with the spread of the stand condition being the largest (*SD* Latitude = 16.1°), the sit condition in the middle (*SD* Latitude = 15.9°), and the smallest in the swivel condition (*SD* Latitude = 13.7°); with significant differences between the sit and swivel conditions (*p* < .001), between the swivel and stand conditions (*p* < .001), but not between the sit and stand conditions (*p* > .05).

If one compares the latitude distributions of the eyes (Fig 3) and the head (Fig 4) one might note that unlike eyes-in-space, the head distributions are above the equator. This above-equator bias was significant for each of the postures: Sit condition M = 15.9°, *t*(26) = 23.5, *p* < 0.001, Cohen’s **d* *= 4.53; Swivel condition M = 13.7°, *t*(26) = 21.3, *p* < 0.001, Cohen’s *d* = 4.10; and S*t*and condition M = 16.1°, *t*(26) = 18.7, *p* < 0.001, Cohen’s *d* = 3.60.

**Fig 5:**
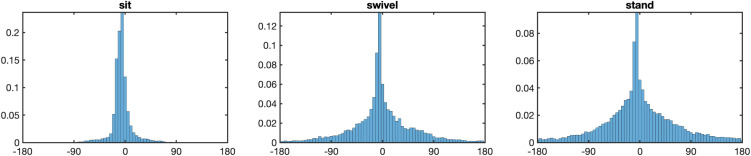
Longitude distributions of torso in space. *Note.* Longitude distributions of torso for posture conditions sit (left), swivel (middle) and stand (right).

Up until now we have simply been concerned with the position of the eyes and head relative to the sphere. Fig 5 presents the rotation of the torso relative to the virtual sphere along the horizontal as rotations in other directions were negligible. From these plots, it is evident that when participant’s mobility is unconstrained in the stand condition, or when they can swivel their chair, they move their torsos to a significantly greater extent than when they are sitting in a stationary chair (*SD* Sit = 11.5°, *SD* Swivel = 46.9°, *SD* Stand = 56.4°). These differences were confirmed by an ANOVA over postures, *F*(1.90, 49.51) = 84.56, *p* < .001, $\eta_{G}^{\text{2}}=\text{0}.\text{4}\text{9}\text{7}$, and Bonferroni-corrected post-hoc comparisions indicating significant differences in torso rotation between the Sit and Swivel conditions (*p* < .001), between the Sit and Stand conditions (*p* < .001), and between the Swivel and Stand conditions (*p* < .05).

To date the reference frame for the analyses has been the virtual sphere. What these analyses have shown is that the eyes, head, and torso move primarily horizontally when exploring the visual scenes, with the head positioned about 13.5–16° above the sphere’s equator. Standing and swivelling postures are generally very similar to one another in terms of the amount of a scene people explore, where they look, and how they look there with regard to moving the eyes, head, and torso. Both these postures exceed the amount and range of movement of the eyes, head, and torso when compared to sitting in a stationary chair. We now turn to examine how these spatial movements operate with regard to one another.

### Combined movements of the eyes, head, and torso

To consider the relative movement of the different effectors we begin by looking at the relationship of the torso relative to the chair in the sitting conditions, then the movement of the head relative to the torso across all postures, and finally the movement of the eyes relative to the head in all three postures. In this way we can understand the nested hierarchy of motion that occurs for each of the effectors as a function of the three different postures.

### Torso relative to the chair

**Fig 6:**
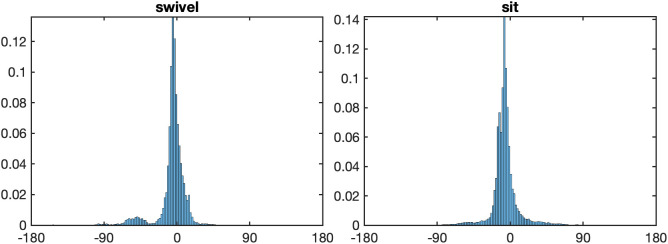
Distribution of torso longitudes relative to chair (for postures swivel and sit). *Note.* Left: Distribution of torso longitudes relative to chair rotation in the swivel condition. Right: Distribution of torso longitudes in the sit condition, repeated from Fig 5.

Fig 6 shows the distribution of the torso longitude relative to the chair in the swivel condition, as well as the torso longitude in the sit condition (repeated from Fig 5 for comparison). The spread of the torso relative to the chair in the swivel condition (*SD* longitude = 19.4°) is similar to the spread of the torso in space in the sit condition (*SD* longitude = 11.5°) shown in Fig 6. Together, the results indicate that the torso is rotated only minimally while sitting in a chair.

### Head relative to torso

**Fig 7:**
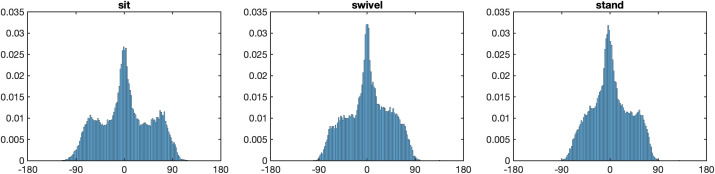
Distribution of head relative to torso. *Note.* Distribution of head longitudes relative to the torso, for postures sit (left), swivel (middle) and stand (right). Note the two side peaks in the distribution of the sit conditions, which do not exist in the other two conditions.

Fig 7 shows the distribution of the head longitude relative to the torso in the three posture conditions. There are several interesting characteristics of these distributions. First, due to physical limitations, the neck can be rotated in a range of approximately [−80°, 80°]. Second, maximal rotation of the head in space can be achieved through rotation of legs, hips, torso, and neck in the stand condition, through rotation of chair, hips, torso and neck in the swivel condition, but only through rotation of the torso and the neck in the sit condition. For this reason, extreme rotation of the head should occur more frequently in the sit condition than in the other two conditions. An inspection of the histograms in Fig 7 shows that this is indeed the case: In the sit condition, there are two side peaks near about ±70°, which are missing in the swivel and the stand conditions.

### Eyes relative to the head

**Fig 8:**
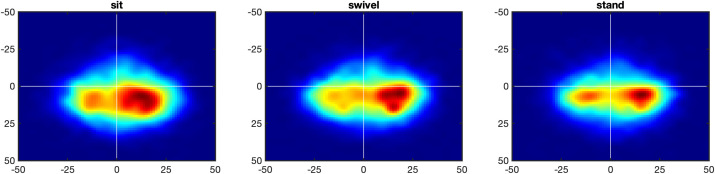
Heatmap of eyes relative to head (eyes-in-head). *Note*. Heatmaps of eyes-in-head points in the longitude range [– 50°, 50°] and the latitude range [– 50°, 50°], with white lines showing 0° longitude and latitude, for posture conditions sit (left), swivel (middle) and stand (right). In all conditions, there are two peaks, one to the left and the other to the right of the head-defined center.

Because it is better illustrated with heatmaps than with frequency distributions, Fig 8 shows the heatmaps of the eyes relative to the head. Statistical analyses shows that posture does not affect the longitude distribution, *F*(1.93, 50.26) = 2.23, *p* > .05, $\eta_{G}^{\text{2}}=\text{0}.\text{0}\text{0}\text{9}$, but the latitude distribution, *F*(1.93, 50.26) = 6.88, *p* < .005, $\eta_{G}^{\text{2}}=\text{0}.\text{0}\text{2}\text{9}$. Further, the means of the eyes-in-head distributions are significantly below the equator for all three conditions, for the sit condition at *M* = −10.7°, *t*(26) = 33.3, *p* < .001, Cohen’s *d* = 6.41, for the swivel condition at *M* = −9.9°, *t*(26) = 29.0, *p* < .001, Cohen’s *d* = 5.58, and for the stand condition at *M* = −10.2°, *t*(26) = 29.3, *p* < .001, Cohen’s *d* = 5.65. This below-equator bias of eyes-in-head is matched approxima*t*ely to the above-equator bias of the head posi*t*ions (see Fig 4), and the two combine into an equator-bias of eyes-in-space (see Figs 2 and 3).

**Fig 9:**
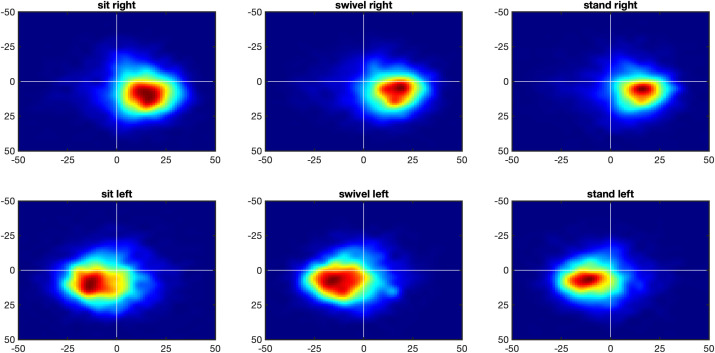
Heatmap of eyes-in-head for head movements to the right and left. *Note*. Heatmaps of eyes-in-head points in the longitude range [– 90°, 90°] and the latitude range [– 90°, 90°], with white lines showing 0° longitude and latitude, for posture conditions sit (left), swivel (middle) and stand (right). Top panel: If the head is moving to the right the eyes-in-head points are to the right of the head-defined center. Bottom panel: If the head is moving to the left the eyes-in-head points are to the left of the head-defined center.

The second interesting characteristic of the eyes-in-head distributions is the bimodality of the distributions, with one peak to the east and the other to the west of the of the head-defined zero-meridian. As explained in [23], the eyes lead the head in almost all cases of panoramic viewing. Thus, if the head moves towards the right, the eyes tend to be to the right of the head position (Fig 9, top row), and conversely, if the head moves towards the left, the eyes tend to be to the left of the head positions (Fig 9, bottom row). If head movement is ignored the double peak of the eye-in-head heatmap is obtained (Fig 8).

### Temporal relationship of eyes, head, and torso

The previous analyses examined the spatial relationship between the different effectors as a function of the three postures. In the final analysis, we examine their spatio-temporal relationship. For example, do the different effectors move together, simultaneously, as a single unit or do the different effectors move sequentially relative to one another.

**Fig 10:**
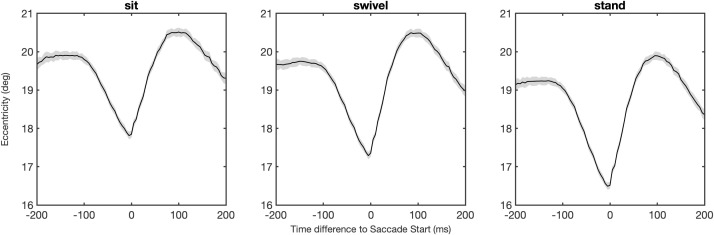
Eyes-in-head eccentricity in temporal relation to saccade starts. *Note*. Average eyes-in-head eccentricity in relation to the saccade starts for the three postures. The grey band indicates the 95% confidence interval of the standard error. Eccentricity diminishes during fixations, reaching a minimum just before a new saccade is initiated (at time difference 0), followed by a rapid increase in eccentricity. The minimal eccentricities very with posture.

We found that eyes-in-head eccentricity varies systematically in temporal relation to fixations and saccades (see Fig 10). During a fixation, eyes-in-head eccentricity diminished slowly, reaching a minimum at the end of the fixation and the beginning of a saccade. In other words, the head movements during fixations lead to the head direction being more closely aligned with eye direction. After the initiation of a saccade, there is a rapid increase of the eyes-in-head eccentricity, after which the cycle repeats. The minimum eccentricity of eyes-in-head at the start of a new saccade varies with posture, for sit *M* = 17.8°, for swivel *M* = 17.4°, and for stand **M* *= 16.5°, a highly significant effect, *F*(2, 84886) = 192.9, *p* < .001, $\eta_{G}^{\text{2}}=\text{0}.\text{0}\text{0}\text{5}$, with significant pairwise differences between postures (all *p*’s < .001).

An analysis of the temporal relation between head and torso rotations showed that the start of torso rotations followed the start of head rotations with a delay that depended on the posture: on average, the head-torso delay was 52.6 ms for sit, 45.8 ms for swivel and 34.0 for stand, *F*(2, 13135) = 4.14, *p* < .02. Post-hoc comparisons showed that the Bonferroni-corrected difference between postures sit and stand was significant (*p* < .02), whereas the other differences were not significant (all *p*’s > .26). This result is consistent with previous results indicating that participants rotate their body in an ordered sequence, with eyes turning first, followed by the head, the torso and then the rest of the body (e.g., [26–30]).

## Discussion

This study systematically investigated how posture -- sitting, standing, or swiveling -- influences visual exploratory behaviour in immersive virtual environments. Our results demonstrate that posture meaningfully alters the coordination and extent of movements across the eyes, head, and torso during panoramic scene exploration. In particular, standing posture facilitated the broadest range of movement and the greatest scene coverage, while fixed sitting posture imposed the most constraints, limiting both the spatial extent and the coordination of gaze-related effectors.

Participants in the standing condition exhibited significantly more fixations and a broader spatial distribution of eye, head, and torso movements. Interestingly, the swivel condition often closely approximated the standing condition in terms of movement patterns, suggesting that freedom to rotate -- rather than upright posture per se -- is a critical factor in promoting naturalistic visual exploration. The fixed sitting condition, by contrast, constrained head and torso movements, resulting in reduced scene coverage and a compensatory increase in eye-in-head movements.

At a more detailed level, the analysis of the exploratory patterns of the scene analysis showed that there were substantial differences in the total distance participants’ gaze moved across the scenes in each trial, small in the sit condition, intermediate in the swivel condition and large in the stand condition. The percentage of scenes covered by fixations confirmed these results with small, but significant differences between the postures, with coverage smallest in the sit condition, intermediate in the swivel condition and largest in the stand condition.

We then analyzed the heatmaps/frequency distributions of eyes-in-space, head-in-space, and torso-in-space. The results were not only consistent with the analysis of the exploratory scene analysis patterns, but also with results obtained previously in the literature (e.g., [8,11,23]). Posture affected the horizontal spread of eyes-in-space, head-in-space, and torso-in-space in the same way, with the spread being smallest in the sit condition, intermediate in the swivel condition, and largest in the stand condition. Posture effects were somewhat less consistent with respect to the vertical spread, which was largest in the stand condition.

These results align with and extend Sidenmark and Gellersen’s findings [14], who showed increased reliance on torso movement when participants stood. Unlike their sparse-target paradigm, our use of relatively rich, immersive 360° scenes mimics real-world exploration and reveals how posture modulates exploratory strategies in environments without predefined targets. Furthermore, while Sidenmark and Gellersen did not include a swivel chair condition, our inclusion of it helps parse apart the influence of posture from the degrees of movement freedom -- a key contribution.

Our findings also highlight the nested and coordinated nature of visual orienting: head movements realign during fixations to reduce eye eccentricity, with the eyes leading the head prior to saccades. This pattern echoes the previous reports by Bischof and colleagues [8,9] but extends their work by emphasising the critical role of posture -- sitting, standing, or swiveling -- which they and others had previously ignored. Collectively, the present study provides strong empirical support for theories of embodied vision, in which visual orientation emerges from the dynamic interplay of multiple effectors.

Our results showed that, in the sit and swivel conditions, participants rotated their torso only minimally with respect to the chair (see Fig 6). In contrast, lateral head rotation was much larger and was influenced by posture, with extreme head rotations occurring more frequently in the sit condition than in the other two conditions, most likely because head rotation was used to compensate for minimal torso rotation in this condition. This emphasizes the crucial role of head rotation (rather than torso rotation) in determining the field of view. Further, the results showed that the horizontal rotation of the eyes-in-head remains unaffected by posture. This finding reenforces the vital role of the head in determining the field of view and supporting oculomotor selection of items in the environment.

In the final analysis, we found that eyes-in-head eccentricity varies systematically in temporal relation to fixations and saccades (see Fig 10). During fixations, the head continues to move slowly to diminish eyes-in-head eccentricity, reaching a minimum at the end of the fixations and the beginning of new saccades. This finding is consistent with previous results indicating that participants rotate their body in an ordered sequence, with eyes turning first, followed by the head, the trunk and then the rest of the body (e.g., [26–30]). The results also indicated that the minima of the eyes-in-head eccentricity were affected by posture, largest in the sit condition, intermediate in the swivel condition, and smallest in the stand condition. This suggests that eye-in-eccentricity may be affected by the energetic effort, with which torso and head can be adjusted to new positions.

From a broader perspective, our results underscore the importance of accounting for posture in VR-based studies of gaze behaviour. Differences in chair type or movement constraints are not trivial -- they directly influence the recruitment of eye, head, and torso movements, and hence affect what participants see and how they see it. This has implications not only for research design but also for practical applications of VR in fields like education, training, or rehabilitation, where movement affordances may vary substantially.

In addition to these contributions, our study raises several opportunities for future research. Our participant sample was drawn from a university population that was heavily skewed towards females (N = 20), with too few males (N = 7) to support a meaningful analysis of sex differences. Given that such differences in spatial behaviour have been reported in the literature and can persist in VR [31], it remains to be determined whether and how our findings generalize to more diverse populations. Nevertheless, our findings that standing and swivel postures enable broader, more coordinated visual exploration in VR environments, add to the growing body of evidence highlighting the importance of movement in clinical rehabilitation. This is further supported by systematic evidence showing that VR interventions enhancing postural freedom can improve balance and mobility outcomes across diverse clinical populations [32], including stroke survivors [33,34] and individuals with Parkinson’s disease [35].

The present work also focuses exclusively on movement data, without including usability or subjective experience measures such as simulator sickness, fatigue, and immersion. Anecdotally, none of the participants reported experiencing motion sickness, and the study duration was relatively short (45 minutes in total, with 19–24 minutes of VR testing), making fatigue unlikely to be a major contributor. Scene content, while diverse, did not produce meaningful variation in exploration behaviour. However, because no task performance measures were included, it remains unclear whether differences in posture influence outcomes like memory and attention, which have been linked to physical and cognitive effort (e.g., [36,37]). In applied contexts, such as gaming, education, or training, these factors -- as well as immersion -- become increasingly important and warrant systematic investigation in future work.

The current investigation employs static 360° images (3 degrees of freedom, DoF) preventing translational movement. This constraint may have accentuated horizontal movements. Similarly, the stimuli were only visual. Future research with 6 DoF will need to be conducted to verify whether the observed pattern persists and extends to dynamic stimuli and the inclusion of nonvisual (e.g., audio) stimulation.

In closing, the present study sought to better understand how different postures influence visual behaviour in immersive virtual environments. Our findings advance the field’s understanding of how physical posture shapes the biomechanics and strategies of visual exploration in VR. By disentangling the roles of sitting, standing, and rotating, we provide compelling evidence that posture is not merely a contextual variable but a core determinant of how vision unfolds in time and virtual space.
